# A new species of Metopiinae (Hymenoptera, Ichneumonidae) parasitizing lepidopteran larvae in China

**DOI:** 10.3897/zookeys.572.8031

**Published:** 2016-03-15

**Authors:** Ying Zhang, Mao-Ling Sheng, Zi-Cheng Xiong

**Affiliations:** 1 General Station of Forest Pest Management, State Forestry Administration, Shenyang 110034, P.R. China; 2 Forest Pest Control and Quarantine Station of Etuoke, Inner Mongolia Autonomous Region 016100, P.R. China

**Keywords:** *Trieces*, new species, *Bazaria turensis*, Pyralidae, Psychidae, China

## Abstract

A new species of Metopiinae, *Trieces etuokensis* Sheng, **sp. n.**, is described and illustrated. Specimens were reared from two species of Lepidoptera: *Bazaria turensis* (Ragonot, 1887) (Pyralidae) from Balong, Dulan, Qinghai Province, and an unidentified psychid (Psychidae) from Mukainor, Etuoke, Inner Mongolia Autonomous Region, China. The new species is characterized by a yellow face and clypeus, fore and middle femora and hind tibia mainly black, antennae slightly longer than head and mesosoma combined, with 17 flagellomeres, occipital carina entirely absent, and the hind femur being compressed, 2.5 times as its long as maximum width.

## Introduction

*Trieces* Townes, 1946 (Hymenoptera, Ichneumonidae, Metopiinae) comprises 68 species (Tolkanitz 2010, Yu et al. 2012), of which 13 are from the Eastern Palaearctic Region (Tolkanitz 2010). No species of *Trieces* have been reported from China to date. The genus is characterized mainly by areolet absent, mesopleural suture indistinct or absent, lateral carina of first tergite weak or obsolescent, second tergite with complete median and sublateral longitudinal carinae, basal portion of sublateral longitudinal and median carinae of third tergite present, and epipleura of third to fifth tergites almost absent (Townes 1971, Gauld et al. 2002, Tolkanitz 1987, 2010).

The known hosts of *Trieces* Townes mainly belong to the lepidopteran families Elachistidae (Bradley 1974), Geometridae (Petrice, et al. 2004), Tortricidae (Walley 1969) and Yponomeutidae (Gencer 2003, Yu et al. 2012).

Herein one new species of *Trieces* is reported, reared from the pupae of *Bazaria turensis* (Pyralidae) and an unidentified psychid (Psychidae).

## Materials and methods

**Figure 1.**
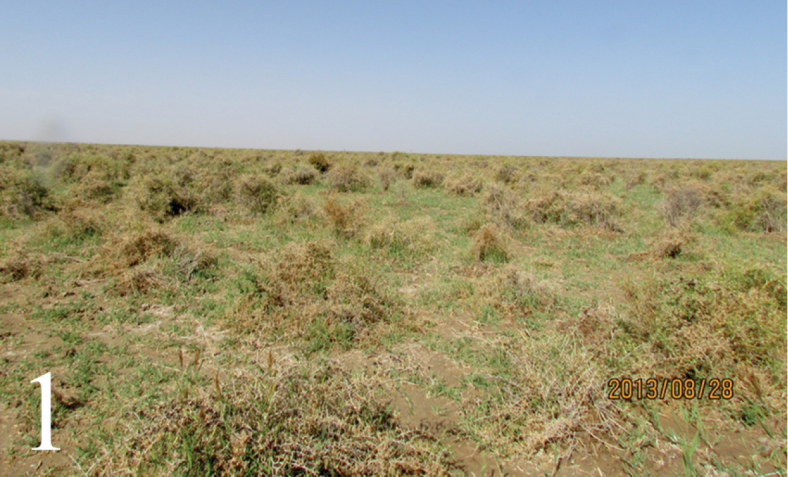
Habitat of *Trieces etuokensis* Sheng, sp. n. The shrubbery in Balong, Dulan County, Qinghai Province.

Mature larvae of the host, *Bazaria turensis* (Ragonot, 1887) were collected on 28 August 2013 in a forest from where there had been an outbreak lasting at least three years, and brought to the laboratory. The forest is a shrubbery (Fig. 1) composed of *Nitraria tangutorum* Bobrov, Lycium chinense Miller var. potaninii (Pojarkova) A.M. Lu and *Kalidium foliatum* (Pallas) Moquin-Tandon, located in Dulan County, 36°09.65'N; 97°27.42'E, Qinghai Province. Mature larvae were maintained in a nylon cage at room temperature, and after pupating were stored individually in glass tubes (60 × 6 mm) with a piece of filter paper dipped in distilled water to maintain moisture and plugged tightly with absorbent cotton. After the emergence of moths and parasitoids was complete, remaining pupae were dissected to record their condition (i.e. status of moths, and parasitism).

**Figure 2.**
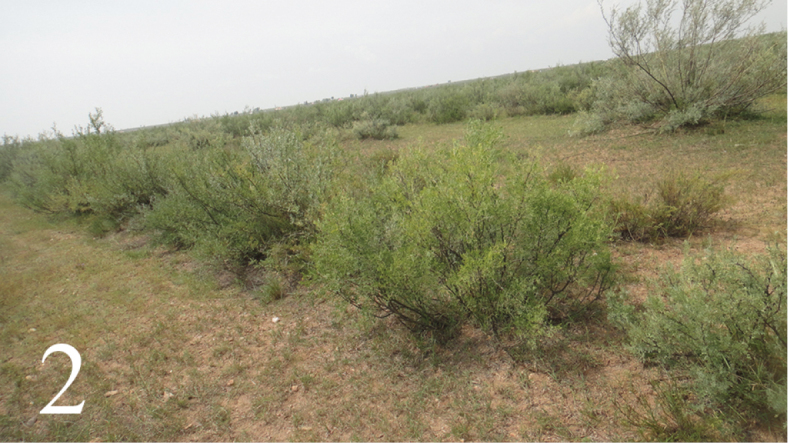
Habitat of *Trieces etuokensis* Sheng, sp. n. The scrub in Mukainor, Etuoke, Inner Mongolia Autonomous Region.

Mature larvae of the psychid moth were collected in 16 September 2014 from a scrub where there had been an outbreak lasting at least fourth years, and brought to the laboratory. The scrub (Fig. 2) is composed of *Caragana intermedia* Kuang & H.C. Fu and located in Mukainor, 39°33.71'N; 108°40.24'E, Etuoke, Inner Mongolia Autonomous Region.

Images were taken using a Leica M205A Stereomicroscope with LAS Montage MultiFocus. Morphological terminology is mostly based on Gauld (1991).

Type specimens are deposited in the Insect Museum, General Station of Forest Pest Management (GSFPM), State Forestry Administration, People’s Republic of China.

## Results

### Trieces

Taxon classificationAnimaliaHymenopteraIchneumonidae

Townes, 1946

Trieces Townes, 1946. Boletín de Entomologia Venezolana, 5:60. Type-species: Exochus texanus Cresson.

#### Diagnosis.

Interantennal process forming a triangular projection in front of antennal sockets. Anterior spur of mid tibia 0.25× to 0.65× length of posterior spur. Mesopleural suture indistinct or absent. Areolet absent. Lateral carina of first tergite weak or obsolescent. Second tergite with complete median and sublateral longitudinal carinae. Basal portion of sublateral longitudinal and median carinae of third tergite present. Epipleura of third to fifth tergites almost absent.

In Tolkanitz’s (2010) key to the species of Palaearctic fauna, the new species can be inserted as follows:

**Table:** 

14 (15)	Head in dorsal view widened posteriorly behind eyes. Occipital carina obsolete. Metapleura as in Figs 3, 15. Face and clypeus of female reddish brown (Figs 5, 4). 2.7. (Male unknown). Russia (Amurskaya Province)	***Trieces femoralis* Tolkanitz**
15 (14)	Head in dorsal view not widened posteriorly behind eyes, parallel-sided. Metapleura as in figs 3, 16. Occipital carina absent. Face, clypeus, and genae of female yellow	**15(a, b)**
15a (15b)	Antenna not longer than head and mesosoma combined; fore wing with vein 1cu-a slightly distal of 1-M, nearly interstitial; length of hind femur 2.2× its width; hind tibia reddish yellow; antennae reddish yellow. (Male unknown). Mongolia	***Trieces pumicatus* Tolkanitz**
15b (15a)	Antenna longer than head and mesosoma combined; fore wing with vein 1cu-a distinctly distal of 1-M, distance between them 0.4× length of 1cu-a; hind femur distinctly compressed, 2.5× as long as maximum width; hind tibia mainly black; basal ventral profile of antennae red brown, basal dorsal profile blackish brown; apical portion brownish black. China (Inner Mongolia Autonomous Region)	***Trieces etuokensis* Sheng, sp. n.**

### Trieces etuokensis

Taxon classificationAnimaliaHymenopteraIchneumonidae

Sheng sp. n.

http://zoobank.org/23C8E597-4794-4069-B60C-665573C8159C

Figs 3–5 , 6–12

#### Etymology.

The specific name is derived from the holotype locality.

#### Material examined.

Holotype female reared from pupa of unidentified psychid moth on 27 October 2014, CHINA: Mukainor, 1476m, Etuoke, Inner Mongolia Autonomous Region, Mao-Ling Sheng. Paratypes: 1 male, same data as holotype except 24 October 2014. 1 female reared from pupa of *Bazaria turensis* (Ragonot, 1887) on 2 October 2013, China: Balong, 2857m, Dulan, Qinghai Province, Mao-Ling Sheng.

#### Diagnosis.

Malar space approximately as long as basal width of mandible. Ocular-ocellar line as long as diameter of posterior ocellus. Antenna slightly longer than head and mesosoma combined, with 17 flagellomeres. Occipital carina entirely absent. Metapleuron shiny, impunctate, lower posterior portion with distinct wrinkles. Hind femur compressed, 2.5× as long as maximum width. Face and clypeus yellow. Fore and middle femora and hind tibia mainly black.

#### Description.

**Female**. Body length approximately 2.8 mm. Fore wing length approximately 2.2 mm.

***Head.*** Inner margins of eyes distinctly convergent downwards. Face (Fig. 4) with dense fine punctures, distance between punctures 0.2–0.5× diameter of puncture. Clypeus with denser punctures than that of face, distance between punctures approximately 0.2× diameter of puncture, apical portion slightly concave; apical margin almost truncate. Mandible small, weakly narrowed toward apex; upper tooth distinctly longer than lower tooth. Malar area flat, with fine punctures. Malar space approximately as long as basal width of mandible. Gena in dorsal view approximately as long as width of eye, almost smooth, with sparse, indistinct fine punctures, scarcely convergent posteriorly. Vertex (Fig. 5) and frons almost shiny, with indistinct fine punctures. Postocellar line 1.2× as long as ocular-ocellar line. Ocular-ocellar line approximately as long as diameter of posterior ocellus. Upper portion of frons slightly convex, lower portion slightly concave. Antenna (Fig. 6) slightly longer than head and mesosoma combined, with 17 flagellomeres; each flagellomere longer than wide. First flagellomere 2.2× as long as maximum width, 1.1× as long as third flagellomere. Occipital carina absent.

**Figures 3–5:**
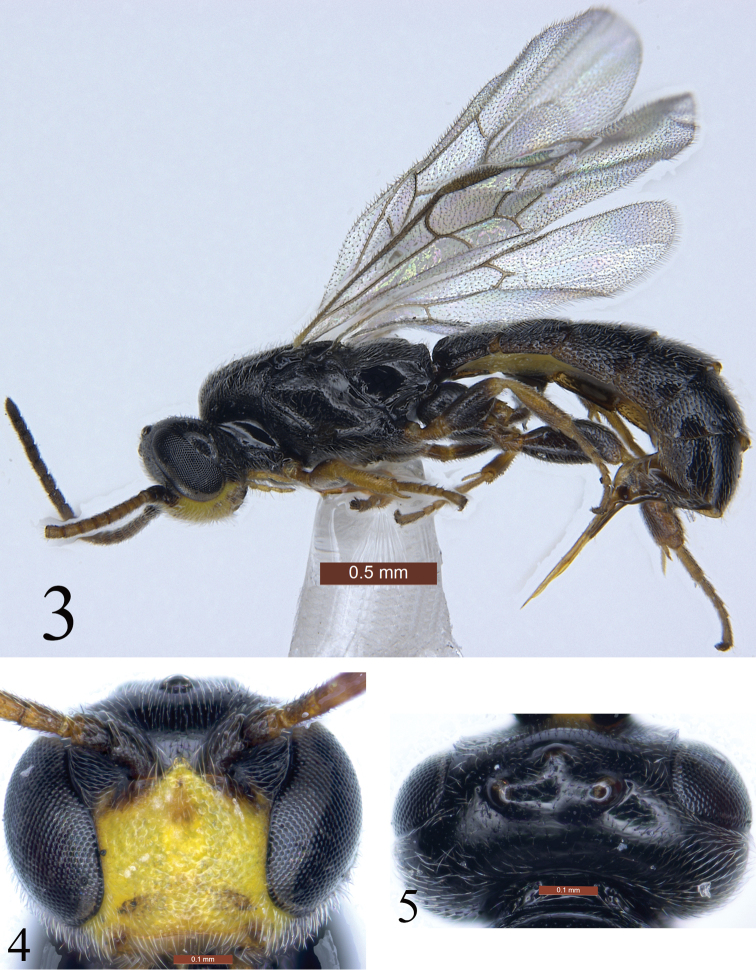
*Trieces etuokensis* Sheng, sp. n. Holotype. Female **3** Habitus, lateral view **4** Head, anterior view **5** Head, dorsal view. Scale bars: 0.5 mm (**3**); 0.1 mm (**4, 5**).

**Figures 6–12.**
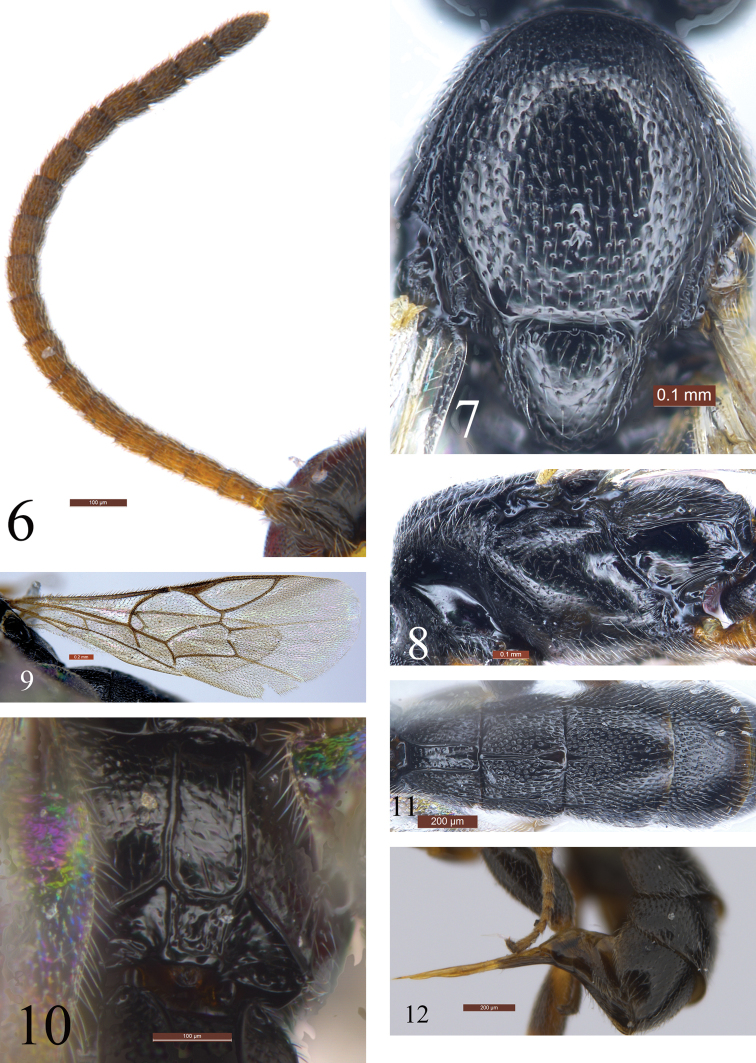
*Trieces etuokensis* Sheng, sp. n. Holotype. Female **6** Antenna **7** Mesoscutum and scutellum **8** Mesosoma, lateral view **9** Fore wing **10** Propodeum **11** Tergites 1-4, dorsal view **12** Apical portion of metasoma, lateral view. Scale bars: 0.1 mm (**6, 7, 8, 10**); 0.2 mm (**9, 11, 12**)

***Mesosoma.*** Lateral concavity of pronotum smooth, shiny, upper-posterior portion slightly rough, with dense shallow fine punctures. Epomia indistinct. Mesoscutum (Fig. 7) smooth, shiny, anterior portion slightly convex, posterior flat; with dense distinct punctures, distance between punctures 0.2–2.0× their diameter. Notaulus absent. Scutellum almost flat, shiny, with sparse, indistinct, fine punctures; lateral carina reaching apex. Postscutellum so short it resembles a transverse carina. Mesopleuron (Fig. 8) with sparse fine punctures. Speculum with fine indistinct punctures. Mesopleural fovea vestigial. Upper end of epicnemial carina reaching anterior margin of mesopleuron, at dorsal 0.75 of posterior margin of pronotum. Metapleuron almost flat, shiny, postero-dorsal portion with few hairs, lower posterior portion with distinct wrinkles. Juxtacoxal carina strong. Wings (Fig. 9) slightly infuscate. Fore wing with vein 1cu-a strongly inclivous, distal to 1-M by about 0.4× length of 1cu-a. 2m-cu straight, distinctly inclivous, distal to 2rs-m by about 1.4× length of 2rs-m. Hind wing vein 1-cu 2.0× as long as cu-a. Hind femur compressed, 2.5× as long as maximum width. Hind tibia gradually widened apically. Posterior spur of hind tibia approximately 2.75× as long as anterior spur. Ratio of length of hind tarsomeres 1:2:3:4:5 is 2.8:1.2:1.0:0.8:1.6. Propodeum (Fig. 10) with strong apical transverse and complete longitudinal carinae. Pleural areas with dense, distinct, fine punctures. Area petiolaris with longitudinal wrinkles. Remainder of propodeum smooth, shiny, with indistinct fine punctures. Propodeal spiracle small, circular.

***Metasoma*** (Figs 11, 12). First tergite approximately 0.75× as long as apical width, with dense punctures; median dorsal, dorsolateral, ventrolateral and sublateral longitudinal carinae complete; apical half with distinct median longitudinal carina. Second tergite (Fig. 11) 1.27× as long as apical width, densely punctate, median and sublateral carinae complete. Third tergite 0.78× as long as apical width; basal 0.7 with dense punctures, apical 0.3 with sparser punctures and smoother than basal 0.7; basal 0.4 with median longitudinal carina; basal 0.3 with lateral longitudinal carinae. Basal 0.6 of fourth tergite densely punctate, apical 0.4 shiny, gradually impunctate. Basal 0.4 of fifth tergite with dense punctures, apical 0.6 gradually impunctate. Ovipositor (Fig. 12) tapered from base to apex, with a large, shallow notch.

***Color*** (Fig. 3). Black, except as follows. Face (Fig. 4) except upper median light brown spot, malar area and clypeus yellow. Maxillary and labial palpi yellow brown. Anterior profile of pedicel dark brown; basal ventral profile of antenna red-brown, basal dorsal profile darkish brown; apical portion brownish black. Anterior and posterior profiles of fore femur, tibia and tarsus, basal and apical portions of mid tibia, mid tarsus, all trochanters more or less, tegula brown to dark brown. Pterostigma (Fig. 9) blackish. Wing veins dark brown.

**Male.** Body length approximately 3.1 mm. Fore wing length approximately 2.5 mm. Antenna with 22 flagellomeres. Very similar to the female, except with hind first tarsomere yellow, apical portion pale brown yellow.

#### Hosts.

One female was reared from pupa of *Bazaria turensis* (Ragonot, 1887) (Lepidoptera: Pyralidae). One female and one male were reared from unidentified species of Psychidae (Lepidoptera) collected as mature larvae but details of development and emergence unknown.

#### Host plants.

*Caragana intermedia* Kuang & H.C. Fu (Leguminosae), *Nitraria tangutorum* Bobrov (Zygophyllaceae), *Kalidium foliatum* (Pallas) Moquin-Tandon (Amaranthaceae).

#### Remarks.

This new species is similar to *Trieces pumicatus* Tolkanitz, 2010 and can be distinguished from the latter by the following combination of characters: antenna slightly longer than head and mesosoma combined, with 17 flagellomeres (female); fore wing with vein 1cu-a distinctly distal of 1-M, distance between them 0.4× length of 1cu-a; hind femur compressed, 2.5× as long as maximum width; lateral longitudinal carinae of tergite 3 distinct on anterior 0.3; fore and middle femora, hind tibia mainly black; basal ventral profile of antennae red brown, basal dorsal profile darkish brown; apical portion brownish black. The same characters for *Trieces pumicatus* Tolkanitz are as follows: antenna not longer than head and mesosoma combined, with 20 flagellomeres (female); fore wing with vein 1cu-a slightly distal of 1-M, nearly interstitial; length of hind femur 2.2× its width; lateral longitudinal carinae of tergite 3 vanishing behind its middle; fore and mid legs, hind tibia reddish yellow, fore and mid femora slightly darkened on outer side; antenna reddish yellow.

## Supplementary Material

XML Treatment for Trieces

XML Treatment for Trieces etuokensis
